# Challenges of using air conditioning in an increasingly hot climate

**DOI:** 10.1007/s00484-017-1493-z

**Published:** 2017-12-30

**Authors:** Karin Lundgren-Kownacki, Elisabeth Dalholm Hornyanszky, Tuan Anh Chu, Johanna Alkan Olsson, Per Becker

**Affiliations:** 1 Department of Design Sciences, Lund University, 221 00 Lund, Sweden; 2 Department of Architecture, Lund University, Lund, Sweden; 3 Centre for Environment and Climate Research, Lund University, Lund, Sweden; 4 Division of Risk Management and Societal Safety, Lund University, Lund, Sweden

**Keywords:** Air conditioning, Climate change, Urban areas, Sustainability, Transdisciplinary

## Abstract

At present, air conditioning (AC) is the most effective means for the cooling of indoor space. However, its increased global use is problematic for various reasons. This paper explores the challenges linked to increased AC use and discusses more sustainable alternatives. A literature review was conducted applying a transdisciplinary approach. It was further complemented by examples from cities in hot climates. To analyse the findings, an analytical framework was developed which considers four societal levels—individual, community, city, and national. The main challenges identified from the literature review are as follows: environmental, organisational, socio-economical, biophysical and behavioural. The paper also identifies several measures that could be taken to reduce the fast growth of AC use. However, due to the complex nature of the problem, there is no single solution to provide sustainable cooling. Alternative solutions were categorised in three broad categories: climate-sensitive urban planning and building design, alternative cooling technologies, and climate-sensitive attitudes and behaviour. The main findings concern the problems arising from leaving the responsibility to come up with cooling solutions entirely to the individual, and how different societal levels can work towards more sustainable cooling options. It is concluded that there is a need for a more holistic view both when it comes to combining various solutions as well as involving various levels in society.

## Introduction

Increasing heat exposure levels are one of the most certain effects of climate change (IPCC 2014) and there is strong evidence of negative health impacts of environmental heat (e.g. Forzieri et al. (2017), Gasparrini et al. (2015), Aström et al. (2015), and Canouï-Poitrine et al. (2005)). Heat exposure is particularly problematic in tropical and subtropical climates (Kjellstrom et al. 2009), although there is a significant heatwave-related mortality risk in warmer temperate climates as well (e.g. Poumadère et al. (2005), Patz et al. (2005), and Kaiser et al. (2007)). Heat exposure is particularly problematic in large cities due to what is referred to as the urban heat island (UHI) effect (Oke 1982; Patz et al. 2005). In addition, areas with already and increasingly hot climates are the areas with high urbanisation rates and population growth (United Nations 2015). This puts escalating numbers of people at risk.

Air conditioning (AC) is promoted as an effective solution to reduce heat stress and protect from heat exposure by providing indoor thermal comfort to avoid heat-related health problems (e.g. Whitman et al. (1997), Chestnut et al. (1998), Davis et al. (2003), Barnett (2007), Bouchama et al. 2007, and Anderson and Bell (2009)). Although there are several good reasons for increased AC use, it is important to question the material, discursive and social aspects of AC. O’Neill (2003) described problems related to the widespread adoption of AC more than a decade ago, and argues against the non-critical approach found in some of the public health and epidemiological research fields that promote AC as the most effective solution (e.g. Whitman et al. (1997), Chestnut et al. (1998), Davis et al. (2003), Barnett (2007), Bouchama et al. (2007), and Anderson and Bell (2009)).

This review paper rests on the premise that there are additional perspectives available than that of solely adopting a technological solution such as AC. Such an updated and expanded overview is particularly pertinent in the light of our increasing cognisance of climate change and the current escalation of AC use. Dahl (2013) anticipates a tenfold increase in energy demand for cooling by 2050 if the use of AC continues to follow current trends. This increase is expected to be concentrated to the fast growing and dense cities in areas with tropical and subtropical climates (e.g. Parkpoom and Harrison (2008)).

The purpose of this review paper is to explore challenges linked to increased AC use as well as more sustainable cooling options in order to inform future approaches to handling urban heat. To meet this purpose, the paper sets out to answer the following two research questions:

- What are the challenges of increased AC use found in the scientific literature?
- What are the possible alternative solutions to AC use found in the scientific literature?

We also illustrate the findings from the literature review with examples from urban areas in hot climates.

### Analytical framework and methodology

**Fig. 1:**
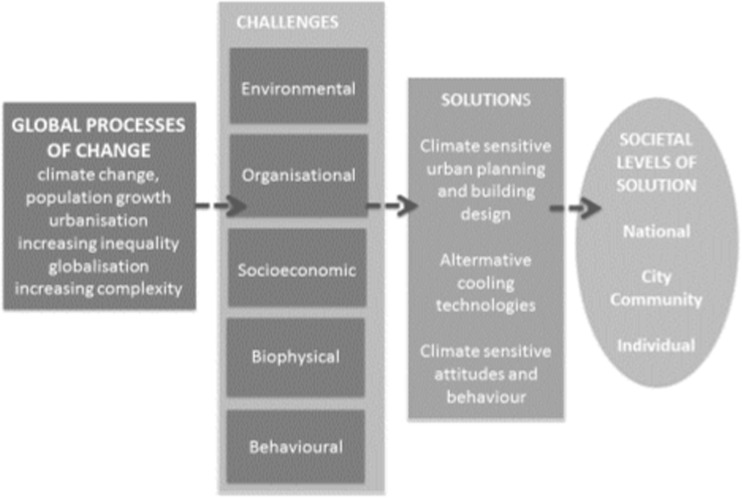
Analytical framework visualising the relation between global processes of change, challenges related to AC use, solutions, and societal levels where solutions are identified

An analytical framework consisting of four parts guided the literature review (see Fig. 1). Inspired by studies on climate vulnerability, we recognised that society is influenced by a set of global processes of change, such as climate change, population growth, urbanisation, increasing inequality, globalisation, and increasing complexity (Becker 2014). We also recognised that society adapts proactively or reactively to the resulting challenges (Adger 2006; Anderson and Woodrow 1989; Kelly and Adger 2000; O’Brien et al. 2007). Inspired by multi-level governance methodology, we assumed that challenges as well as solutions may be produced or addressed at different levels in society. Consequently, we added a spatial dimension to the challenges and structured the discussion according to the level of societal organisation the solution addresses (Fig. 1). Particular attention was given to identifying circular relationships, in which one challenge is reinforced through the feedback from another.

A literature review of peer-reviewed papers explored the challenges of AC use. A literature review is a suitable methodology for the purpose of this paper because it can be used to create an overview of what is known in a specific area, and can add detail and depth to a specific problem (Bryman 2008). The development of search terms for the literature search was carried out in a transdisciplinary setting to ensure a broad identification of challenges and alternative solutions, including environment and sustainability science, architecture and urban planning, social sciences, health, risk management, and thermal environment research. The literature review involved a three-step process:

Step one included the following: (i) the identification of search terms and alternatives to increased AC use, (ii) database searches, (iii) identification of challenges, and (iv) categorisation of challenges in the framework. Step two comprised the use of the search terms and categorisations to investigate what the current literature says about the five identified challenges of increased AC use to handle urban heat: environmental, organisational, socioeconomic, biophysical, and behavioural. In step three, alternative solutions were identified through a second literature search, also of peer-reviewed papers. Solutions were categorised into three broad categories: urban planning and building design, alternative cooling technologies, and attitudes and behaviour. These categories relate to the different societal levels where solutions can be implemented (Fig. 1). This is a novel approach to discussing the role of cooling in a hot climate compared to those provided by the narrower-scope literature currently available.

## Challenges with air conditioning

This part of the literature review is structured according to the five categories of challenges (Fig. 1). The first three categories—environmental, organisational, and socioeconomic—are systemic. The last two—biophysical and behavioural—are individual but intimately related to the societal context and socioeconomic situation of individuals.

### Environmental challenges

The adoption of AC increases the use of electricity or energy. It is currently estimated that the world consumes about one trillion kilowatt hours (kWh) of electricity for AC annually, more than twice the total energy usage of Africa for all purposes (Dahl 2013). The modelling results of Isaac and van Vuuren (2009) show that world energy demand for AC will increase rapidly in the twenty-first century. The increase in the median scenario for AC-induced growth in electricity use is from close to 300 TWh in year 2000, to about 4000 TWh in 2050 and more than 10,000 TWh in 2100 (Isaac and van Vuuren 2009). Widespread AC use places a heavy burden on the electricity distribution system and increases the risk for electricity power cuts (Parkpoom and Harrison 2008). IIASA's Global Energy Assessment report (2012) identifies that the choices of cooling technology will prove increasingly important for the development of energy use. These choices are already causing problems in many parts of the world due to increased electricity use (IIASA 2012).

As the use of AC is resource and energy intensive, it has consequently a potential negative impact on both climate change and the environment in general (Brager et al. 2015). Its impacts on climate change depend on the type of energy source used to produce the cooling. AC significantly increases electricity use (Valor et al. 2001; Crowley and Joutz 2003; Parkpoom and Harrison 2008; Izquierdo et al. 2011; Liu et al. 2011; Rosenthal 2012; Lundgren and Kjellstrom 2013). AC also contributes to the UHI effect and directly affects outdoor thermal comfort on streets through heat ejection (Yahia and Johansson 2013). Elevated urban temperatures, including UHIs, can increase the magnitude and duration of heat waves and cause additional night-time electricity consumption from AC (Kovats and Akhtar 2008). Climate change will create higher outdoor heat exposure levels (IPCC 2014) and if developments follow the current trajectory both in relation to expected temperature rises and the speed of AC adoption, there will be an increased use of AC in urban areas especially in the tropics and subtropics. This will create a negative feedback loop related to energy use and to the UHI effect.

One example where the use of AC is growing rapidly is in South and Southeast Asia (Isaac and van Vuuren 2009). In 2010, the Vietnamese building sector accounted for 20 to 24% of the total national energy use, which is expected to increase significantly, especially due to increased AC use (Nguyen et al. 2011; Le Phan and Yoshino 2010). From 1998 to 2008, the nationwide electricity consumption increased by 400%, in which the electricity consumption for administration and household accounts made up the largest proportion (Nam et al. 2015). This development is mostly driven by income growth and poor building design, but also by increasing heat exposure due to urban growth. It is expected that Hanoians will continue to rely solely on AC for cooling, since it is among the fastest growing AC markets globally and the general level of awareness fails to address problems associated with AC usage (Pham et al. 2014). Furthermore, the current situation with rising temperatures in Hanoi has undermined all attempts to reduce energy consumption, especially for cooling purposes. In fact, a heatwave in 2015 showed a record high 30% increase in maximum use of electricity in Hanoi (Lao Dong News 2015). A number of official statements from Vietnam Electricity have indicated that the need for cooling accounted for the largest part of the increase.

### Organisational challenges

The organisational challenges are related to how we chose and organise supportive services and how we organise and design the development of our cities. When people grow increasingly dependent on AC for cooling their homes, they are not only contributing to increased urban temperatures due to outlet of hot air, but they are also increasing their vulnerability. Building houses and communities that are dependent on AC for coping with heat places them at the mercy of electric power cuts, which may become more common when the use of AC intensifies the stress on the electric distribution system (Parkpoom and Harrison 2008). Increasing dependencies between societal systems, such as systems for electricity production and distribution and ones that provide thermal comfort, are well-known contributors to increased risk and vulnerability, as the growing complexity increases the likelihood that two or more failures interact in ways that are difficult to anticipate (Perrow 2008). A consequence of such dependencies is that it becomes gradually more difficult to overview and manage the multiple layers of risk, as well as the consequences of actual events and decisions to handle them, since effects can spread across these chains of dependencies throughout society (Rasmussen and Svedung 2000).

Being dependent on electricity for cooling is, in other words, making people dependent not only on the functioning of the electricity distribution system as such, but also on all other connected systems. For instance, an increased need for electricity also creates a dependency on global oil and coal prices or on political decisions regarding water resource management, since fossil fuels and hydropower stations are the main energy sources in many countries. In addition, such a society becomes more vulnerable to cyclones and other hazards that may cause major destruction and prolonged power cuts (e.g. Han et al. (2009)). Becoming more dependent on electricity for cooling homes thus locks people into a web of dependencies that ultimately increase their vulnerability to heat.

At the heart of this problem lay, in other words, dependencies through which interconnected effects can cascade and reinforce each other in society (Rinaldi et al. 2001; Little 2002). Such dependencies and slowly evolving increases in societal complexity have been referred to as “creeping dependencies” (Hills 2005), which accumulate and eventually reach a threshold where we lose the oversight and much of our ability to manage risks in our societies.

There are many reasons why societies have developed into highly complex structures but they will not be discussed in this paper. However, according to the literature review, it is obvious that current and previous trends in urban development and design are influencing and worsening the dependency on AC. One of them is the globalisation of urban development and housing ideals/trends, resulting in urban designs and buildings that abandon vernacular building tradition and lack the knowledge of how to adapt to the local climate that has developed over centuries.

This is the case in Hanoi where the “new urban areas” (NUAs) is an urban planning model that has been promoted since the turn of the century to meet the increasing demand for housing (Tran 2008). These master-planned developments at the city’s peripheries are more spread out, which creates a different urban space from the very dense and compact older parts of Hanoi’s inner city. The high-rise apartment blocks and detached houses are built to rely entirely on AC and little attention is paid to build in an energy-efficient manner (Le Phan and Yoshino 2010). The connection between design and the escalation of energy consumption for cooling has been shown to be significant, especially when considering the effects of urbanisation and rising urban temperatures (Nam et al. 2015). The increasing use of AC combined with global trends in urban and building design enforces a growing urban heat vulnerability as well as increased emission of greenhouse gases.

### Socioeconomic challenges

At present, AC is mainly an investment made by individuals and enterprises. Its costs comprise the initial investment, maintenance, and running electricity expenditures. In a household context, AC is generally a local solution addressing the heat problem in one room at a time, which in practice means that a house or apartment without a central cooling system needs several AC systems to be able to cool the whole living area. Central cooling systems exist and are mainly used in office buildings or in the upper range private homes. As the economic burden to install a cooling system lies on the individual household, it is causing heat inequities between richer and poorer segments of society. O’Neill (2003) has shown that improvements of social conditions can reduce the inequalities of heat mortality.

With the improvement of living standards, electrical household appliances become more popular and the household energy consumption increases (Le Phan and Yoshino 2010). This situation can be illustrated by the case of Hanoi, where Le Phan and Yoshino (2010) show that the number and frequency of use of AC units are related to the households’ monthly income and have greater effects on the annual energy consumption than the use of any other household appliance. The electricity consumption of households using AC was 4 GJ higher than of those without (Le Phan and Yoshino 2010). This illustrates that the improvement of living conditions results in lifestyle changes, such as increased AC dependency and thus increased energy consumption. For a high-income family, the investment in one or two AC systems or a central cooling system and the payment of the electricity bill are lesser problems. For a poorer family, buying an AC and paying for the electricity may be impossible.

In addition to economic inequality, there is also a gender aspect to AC use. In Hanoi, for example, AC systems are rarely used in the kitchen where it is particularly warm from cooking activities (Phan and Yoshino 2010). There are also other studies showing gender biases related to the adoption of new technology (e.g. Gasper and van Staveren (2003), Iversen (2003), and Fernandez et al. (2013)). Moreover, males and females do not spend an equal amount of time in each room of the house, and women generally are more engaged in domestic activities than men (Carswell 2012) and spend more time at home. The location of the AC units may, in other words, strengthen inequality both within and between families.

Furthermore, urban residents, especially the urban poor, are vulnerable to heat waves due to sub-standard housing, such as poor roofing and less green surroundings (Harlan et al. 2006). Evidence also indicates that the poorest, often living and working in the urban core, are more susceptible to UHI effects (O’Neill 2003). Hence, being unable to afford the purchase or running costs of AC causes several inequities and human rights challenges including social, economic, and gender-related inequalities.

Moreover, the poorer segments of society are less likely to work in an AC environment, which in a hotter climate exposes this societal group to more heat. Several studies show how heat exposure impacts workers in India (Venugopal et al. 2016; Lundgren et al. 2014; Balakrishnan et al. 2010; Ayyappan et al. 2009). The workplaces studied by Venugopal et al. (2016) had very high heat exposure in the hot season, often reaching the international standard safe work values (ISO 7243:^1989^, see example in the next section) impacting worker’s health and productivity. AC is commonly not used in these workplaces; however, due to the increasing adoption of AC (BIS Research 2015) together with the expected increases in temperature as the result of climate change in the region (IPCC 2013), further impacts on workers’ productivity are expected in addition to adverse health impacts.

### Biophysical challenges

The physiological basis for the effects of heat on humans is well understood (e.g. Burton (1937), Ladell (1955), Budd (1974), Hales and Richard (1987), and Parsons (2003)). Humans are born with a highly specialised complex of thermoregulatory sweat glands and a sensitive control system. However, factors, such as pre-existing disease, clothing, age, gender, heat acclimatisation ability, level of physical activity, and body size, can influence this system. When the ambient temperature reaches or exceeds the human core temperature of 37 °C, there are well-documented physiological effects on the human body, posing risks to some organ systems (Bennett and McMichael 2010). As the core temperature begins to rise, skin blood flow increases and sweating is initiated. At core temperatures beyond 38–39 °C, there is an increased risk of heat exhaustion and beyond these temperatures, heat stroke can occur with a consequent failure of the thermoregulatory system (Jay and Kenny 2010). Health consequences range from dehydration, injuries, and heat fatigue to a higher burden of respiratory and cardiovascular diseases, kidney failure, weakening of the immune system, and finally death (Parsons 2003).

**Fig. 2:**
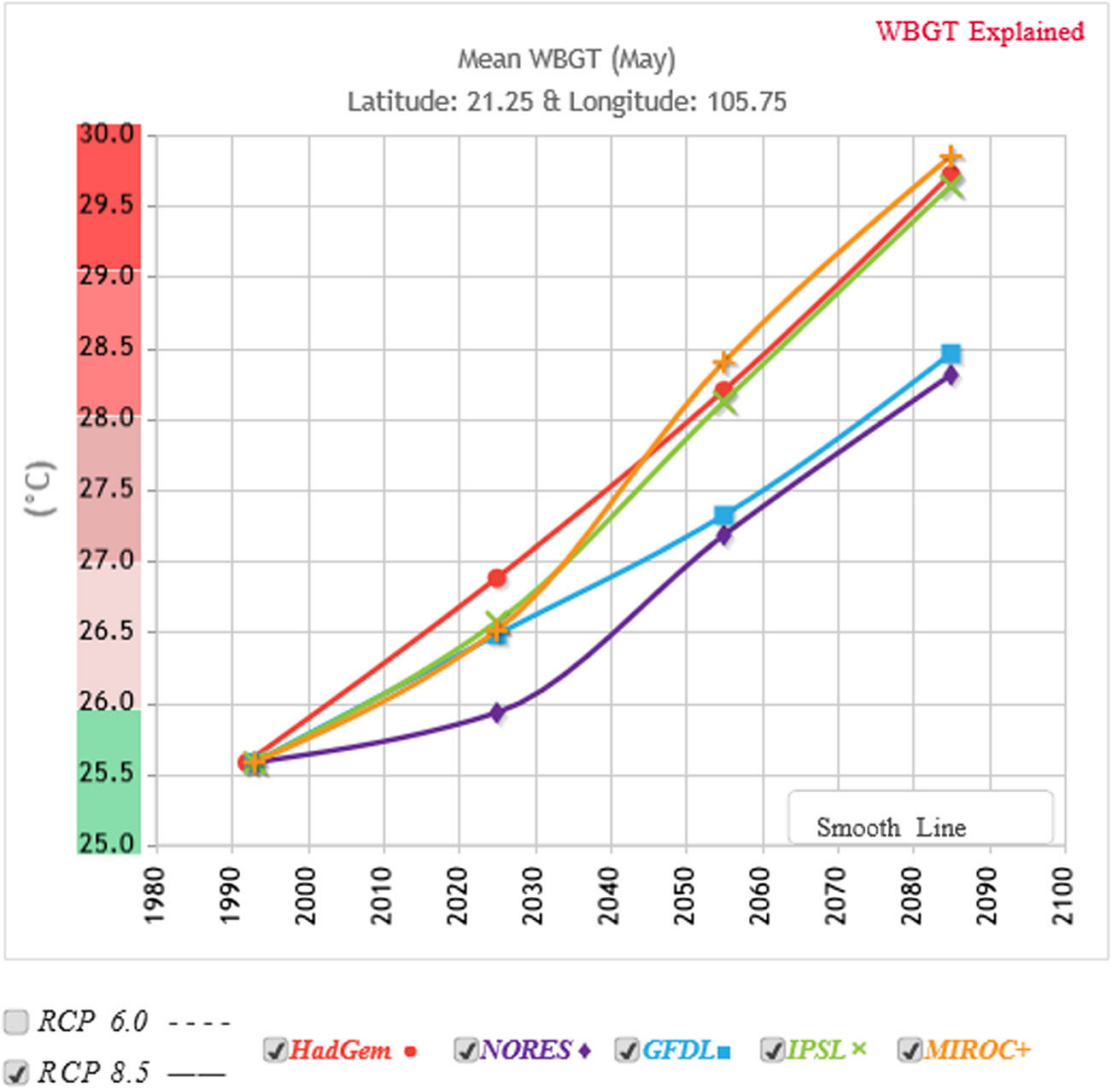
Future heat stress simulations during the month of May in Hanoi, without taking solar radiation into account. Produced by HOTHAPS soft (Kjellstrom et al. 2013; Lemke and Kjellström 2012). The different colours represent the different climate models’ datasets of RCP 8.5 (red—HadGem, violet—NORES, blue—GFDL, green—IPCM, and brown—MIROC+)

One way of measuring heat is by using one of the heat stress indices. The importance of these indices is that the heat stress experienced is related to many environmental factors. One of these indices is the Wet Bulb Globe Temperature (WBGT) widely used in assessments of occupational heat stress (Bernard et al. 2005; Gao et al. 2017 this issue). The ISO standard for WBGT (ISO 7243:^1989^) incorporates environmental temperature, humidity, and solar radiation (Kleim et al. 2002; Gao et al. 2017 this issue). A WBGT of 27 °C is seen as a threshold for the need for actions to protect workers, depending on the intensity of the work and clothing worn (ISO 1989). Assuming that the indoor temperature is similar to outdoor temperatures without AC, the contribution of solar radiation to WBGT needs to be discounted. Using Hanoi as an example for a future potential heat exposures in residential buildings, Fig. 2 shows such an average calculated WBGT heat stress index for the month of May in Hanoi over time based on the IPCC’s representative concentration pathway (RCP) of 8.5 (Climate CHIP 2016; IPCC 2013) using modelling data from the University of East Anglia. All simulations reach the threshold WBGTs of 27 °C or more before 2050, even without the contribution of the UHI effect. This indicates a challenging future for Hanoi. It strongly suggests an increased need to reduce indoor temperatures and if AC is the main available technology, it is likely to be increasingly used.

Humans are able to acclimatise to heat (Parsons 2003). Acclimatisation to a hot environment commonly occurs after 7–14 days of at least 2 hours daily heat exposure (NIOSH 2013). However, physiologically, it is possible for people to lose their heat acclimatisation when spending a majority of their time in AC environments, although the evidence is unclear (Kovats and Hajat 2008). A substantial amount of time spent indoors is required to lose heat acclimatisation (Garrett et al. 2009). Generally, acclimatisation effects are considered to be lost if the heat load has not been experienced for over 2 weeks. Re-acclimatisation depends on individual factors and the extent of time unexposed to heat (Ashley et al. 2015; Cheung and McLellan 1998; Pandolf 1998). Several scholars point out that more research is needed concerning acclimatisation (Pandolf 1998; Aoyagi et al. 1997; Garrett et al. 2009; Lim et al. 1997; Gill et al. 2001; Weller et al. 2007; Wyndham and Jacobs 1957).

### Behavioural challenges

Culturally, AC has brought with it what can be called an encapsulation of the home in warm regions and has led to significant changes in the social geography of the home, as well as the neighbourhood (Wilhite 2009). The adoption of AC is globalised and is occurring at a rapid pace, fostered by the spread of modern building practices and faith in modern technical solutions to achieve indoor thermal comfort. Local knowledge about how to develop a comfortable climate, both indoor and in the neighbourhood, is lost in this process and cooling solutions are increasingly left to technical experts (Wilhite 2009). Wilhite (2009) goes on to argue that the current high demand for AC is socially and technically constructed, originating in the USA, and a part of the global discourse of how a modern house should be built—a development favoured by powerful commercial actors.

Some researchers argue that with increased AC use, people are becoming both physically and mentally dependent and accustomed to cooling, making them more vulnerable to increased urban heat (Nicol and Roaf 2012). As a consequence, traditional vernacular building styles found comfortable by past generations cannot meet current thermal comfort standards (Nicol and Roaf 2012). Moreover, de Dear and Brager (2002) argue that households living year-round with AC are likely to develop high expectations for a cool environment and become dependent on thermal homogeneity within a narrow range of temperatures. Brager et al. (2015) even suggest that as people gradually become reliant on cooling, they will become liable to over-cooling.

Due to a global discourse on the ideal human body, there is also an increasing fixation on eliminating sweat and body odour (Wilhite 2009). Humphreys et al. (2007) discuss a growing global lifestyle and global fashion industry promoting clothing/dress codes that are not in line with global indoor climate requirements, including no sweating (Humphreys et al. 2007). The literature in this research field thus suggests that the globalised perceptions of a modern life is encouraging an increased use of AC. Behavioural and cultural mechanisms are still of dominant importance for daily survival in hot environments (Lundgren et al. 2013), which makes changing individual norms and attitudes vital factors in the escalating demand for cooling technology.

## Alternative or supplementary solutions to air conditioning

This section of the literature review focuses on alternative or supplementary solutions to AC use. The section is structured in three broad categories: climate-sensitive urban planning and building design, alternative cooling technologies, and climate-sensitive attitudes and behaviours.

### Climate-sensitive urban planning and building design

Urban planning is important to address the effects of climate change (UN-Habitat 2007). An unfavourable outdoor and indoor climate can be prevented or mitigated through the application of climate-sensitive urban and building design, which refers to measures that adapt the urban landscape to the site, the region, and the climate (Keitsch 2012). With appropriate urban design, the urban microclimate can be improved and the UHI effect considerably reduced. Urban morphology, in particular the height-to-width ratio of urban street canyons, has a significant influence on air temperature, solar radiation, and wind speed (Johansson 2006). In addition, the orientation of streets and buildings in relation to the prevailing wind directions has a large impact on both outdoor and indoor ventilation (Givoni 1992; Ng 2009). A compact urban design results in considerably less radiation at street level and consequently lower daytime temperatures, which reduces thermal stress compared to a dispersed urban design (Johansson and Emmanuel 2006; Yahia and Johansson 2013, 2014). However, since buildings cannot provide shading at high solar elevations (around noon), overhead shading—either through vegetation or shading devices—is crucial to creating a good microclimate (Emmanuel et al. 2007; Johansson et al. 2013; Yahia and Johansson 2014). Climate-sensitive building design also includes various strategies to maximise ventilation and to minimise solar heat gain, such as proper orientation of the building, adequate design of windows, and use of shading devices and of reflective surface materials (Givoni 1998).

The amount of vegetation affects both air temperatures and radiation in the urban outdoors. In hot and humid climates, plentiful vegetation in the form of large urban parks and gardens reduces urban temperatures considerably (Jusuf et al. 2007; Yahia et al. 2017 this issue). Vegetation can provide multiple positive functions at both building and urban scales, including a reduction of energy use in buildings during cooling periods (Pérez et al. 2014) and improved storm water management (Susca et al. 2011). Street trees, pergolas, etc., are beneficial to create shade in the urban outdoors. A combination of horizontal and vertical green structures is highly recommended to enhance the outdoor thermal environment (Yahia and Johansson 2014; Yahia et al. 2017 this issue). Such green structures have not been considered as an urban feature in the current Hanoi master plan, which does not include enough green areas to mitigate the UHI (Trihamdani et al. 2014). Until the beginning of the 1990s, Hanoi was known as a green city with tree-lined streets and avenues and a good number of public parks, gardens, and small rivulets and lakes (Matsumuto and Almec 2015). During the construction boom in the 1990s, many of the water surfaces were paved or built over and urban green decreased significantly in the city centre (JICA 2007). However, the NUAs have relatively high green space coverage. A UHI simulation study conducted in 2012 (Nam et al. 2015) investigated the cooling effects of the green space network proposed in the Hanoi master plan. The simulated weather data included air temperature, relative humidity, wind speed, wind direction, solar radiation and air pressure. The results showed that high air temperature areas, with temperature of 40–41 °C in the summer, would expand substantially in the planned NUAs. Simulated nocturnal air temperature would increase by up to 3–4 °C and wind speed was weaker than over green spaces. The results also showed that the green strategies proposed in the master plan were able to reduced night-time air temperature within the green areas but could not be expected to cool all of the built areas (Nam et al. 2015). Finally, traditional urban forms and building designs have proven to effectively manage local climate conditions in many countries. Therefore, sustainable and climate-sensitive design requires learning from vernacular ways of building in combination with modern design solutions and technology (Yahia 2014).

### Alternative cooling technologies

At present, there is ongoing research and development of innovative cooling technologies and strategies that could potentially lower energy consumption (Chua et al. 2013; Desideri et al. 2009; Ghazali et al. 2012). District cooling systems and renewable energy AC are potential alternatives to conventional AC (Gang et al. 2015). Examples of district cooling are available, for instance in Singapore, but not very common around the world (Jusuf et al. 2007). Solar cooling and absorption chillers (referring to any conditioning system using passive solar), solar thermal energy conversion, or photovoltaic conversion, are new technologies which have a high potential to replace conventional cooling technology based on electricity (ESTIF 2010). The advantage with solar cooling is that the energy production is renewable and also local which is good for the regional energy supply and for the energy user. Local energy production makes each region more self-sustaining and resilient to power failures (Lundgren and Kjellstrom 2013).

Novel cooling technologies also include personal cooling, such as cooling vests with phase change materials (Gao et al. 2012). Such systems have potential to cool the person’s micro-environment. However, they are often expensive and thus not accessible or seen as a priority by the poor. At an individual level, many people who cannot afford AC are using homemade cooling devices with a similar effect, but built with simple and inexpensive materials, such as fans, ice, and foam boxes. In Hanoi, some of these types of solutions have been commercialised to meet the demands of low-income households. Although such systems are available for the less affluent in society, they have their own environmental impact, as energy and water is needed to produce ice.

### Climate-sensitive attitudes and behaviour

Studies indicate that behavioural changes may be more efficient than physical changes when it comes to reducing energy consumption (Vale and Vale 2009). Thus, a more sustainable urban development with less AC use and less energy consumption does not only require technical solutions or expert knowledge, but also the involvement of civil society (Larsen and Gunnarsson-Östling 2009). It requires efforts from urban citizens to contribute with an adaptive use of the built environment and an adaptive lifestyle, which for instance could mean staying in the shade, having a siesta during the hottest time of the day, or using climate smart clothing.

Mechanical cooling—providing thermal comfort by creating a steady, monotonous environment—has been proven not to be ideal in several cases (Brager et al. 2015). Instead, an adaptive environment with elements of individual control has the potential to provide a superior thermal environment, as it is adapted to the outside heat as well as to the person. Research has shown that when a cool stimulus is applied to a local body part (e.g. hand, head), it serves to reduce whole-body thermal stress (Zhang et al. 2010).

However, many parts of the paradigm of how to build and provide thermal comfort need to change in order for an adaptive environment to become a reality (Chappells and Shove 2005). Several researchers suggest that by actively scrutinising what is perceived as an ideal indoor environment and the associated ways of life, it is possible to develop an understanding of how we can design more sustainable future housing in different climatic conditions (Chappells and Shove 2005). In addition, one has to keep in mind that thermal comfort is individual and can only be understood through a perspective that accounts for the context of historical, technical, and social change (Wilhite 2009).

## Suggested solutions and responsibility at different societal levels

In this final section, we relate the identified solutions to different societal levels. Solutions at the individual level include changes in behaviour, awareness, the deployment of micro-cooling, and personal adaptive strategies. At the community level, there are solutions that can help the most vulnerable, including shared cooled spaces, sharing work and other responsibilities, and investing together in, for example, gardens, houses, and cooling technology. City authorities are responsible for the development of urban space that can handle increasing urban heat, for developing initiatives, and for ensuring that the processes are participatory. The national level is responsible for providing infrastructure (large-, medium-, and small-scale) along with initiatives such as subsidies for sustainable cooling solutions. In addition to these solutions, participation between the actors of one level and the collaboration of actors between levels has been forwarded as being a central means to facilitate learning and increase the implementation and dissemination of new technologies as well as decrease vulnerability (Ahmad and Abu 2015; Bal et al. 2016). Participation can be seen as a cross-cutting solution that is usually used in combination with one or two of the other solutions, either to enhance the uptake of a solution at one level or to create uptake and learning between levels by creating a learning process. Table 1 provides an overview of alternative or supplementary solutions on the different levels.

**Table 1:** Suggested solutions through a division of measures and the societal levels responsible

Measures/level	Climate-sensitive building and urban planning	Alternative cooling technologies	Climate-sensitive attitudes, behaviour, legislation, and education
Individual	Double façade, cross ventilation, orientation of buildings, reflecting surface materials, shading devices, and roof and walls insulation. Green roofs, green walls and shading trees.	Low-tech solutions including fans, self-invented cooling systems using water, ice and fans, desert coolers (a simple mechanical ventilation system based on evaporative cooling), shade structures. High-tech solutions including solar panel-driven AC, absorption chillers, small-scale electricity production (photovoltaic).	Awareness of the impact of heat and how to adapt to it by moving inside the house, climate smart clothing and food, footbath, work/rest regimes, wet towels, frequent showers, pouring of water on roof and floor.
Community	Common indoor and outdoor spaces to be used collectively on hot days. Shade structures.	Local electricity production (windmills, photovoltaic parks). District cooling.	Help to the most vulnerable. Capacity building and risk management on district level.
City	Building orientation adjusted to solar radiation and main wind directions. Protection of existing vegetation, rivers, and lakes. Establishment of new green areas and shade structures.	Municipal infrastructure for renewable energy (windmills, photovoltaic parks). District cooling systems and innovative cooling technologies such as solar panel-driven AC and absorption chillers.	Local laws and regulations for climate-sensitive design. Capacity building for professionals and citizens. Risk assessment initiatives.
National	Developing urban planning regulations to include climate-sensitive design and planning.	National infrastructure for renewable energy supply (large-, medium-, small-scale electricity production).	Capacity building on national level. Subsidies for climate smart solutions. Local laws and regulations for climate-sensitive design. Systems for risk management.

It is clear that AC has its advantages but if we chose to completely rely on it for cooling, it can lead us to a situation with escalating energy consumption, heat stress inequalities, and uncontrollable chains of risks. To address these challenges, a range of actions is required and there is no single solution to provide cooling in a sustainable way due to the complex nature of the problem.

From the analysis, it became clear that there are several challenges related to AC use and all need to be considered in order to gain a more holistic perspective when considering AC adoption. However, the identified challenges and suggested solutions are by no means exhaustive and there is an urgent need for a better and more holistic approach for how to handle our growing urban heat challenge. Different parts and levels of society need to cooperate and new ways of involving civil society must be developed.

A circular learning process between different levels in society is necessary. Instead of leaving all the responsibility for handling heat to the single citizen or household, the responsibility should be more clearly shared. Here, the local and national authorities have to take the lead and at the same time promote engagement and suggestions from civil society. For example, to handle challenges linked to culture and behaviour, civil society can play a role to foster climate-sensitive attitudes. Apart from the individual’s choice to live in a more climate-sensitive way, the community can play an important role in the process of changing citizens’ attitudes and behaviour. Such communities include official and unofficial unions, organisations, and local people. According to Geertman and Le (2008), the involvement and participation of communities also has a positive impact on households’ income and the living environment. This co-operation on the community level could also be the basis for joint investments for cooling systems or energy production. The challenges of unequal access to AC are present and need to be tackled by looking at alternative solutions and ensuring that either the community or the city level is able to protect the more vulnerable segments of society.

When contemplating future directions of thermal comfort, it is also important to reflect on historical ways of handling heat, as centuries of developing vernacular ways of coping and adapting to heat are being lost in a matter of decades or even years. Behavioural and cultural mechanisms are important for everyday coping in hot environments, but the increased use of AC combined with global trends in urban and building design generates increased urban heat vulnerability as well as increased emission of greenhouse gases.

## Conclusions

The purpose of the research presented in this paper was to explore the challenges linked to increased AC use, and to discuss possible alternative or supplementary solutions that could be more sustainable based on a literature review. The results show that there are multiple challenges in relation to AC use that have to be considered. The challenges are not only related to an increase in energy consumption and the associated adverse environmental effects, but to individuals and societies becoming more dependent on AC. This is because frequent use of AC can lead to humans losing the capacity to handle heat both physically and mentally, and thus make them more vulnerable to increasing urban heat. Moreover, increased heat affects people unequally as different socioeconomic segments of society have different capacities for responding to the escalating challenges.

This paper identified several measures to take into consideration to reduce the fast growth of AC use: the implementation of more climate-sensitive building and urban design, the development of more energy-efficient cooling devices that rely on renewable energy and are also affordable for poor people, the promotion of a change towards more climate-sensitive life styles, and the support of co-operative cooling solutions on community and city levels that include active participation in the development of such structures by local communities. In conclusion, there is a need for a more holistic view both when it comes to combining various solutions as well as involving various levels in society.
